# Responsible Reporting: Neuroimaging News in the Age of Responsible Research and Innovation

**DOI:** 10.1007/s11948-015-9684-7

**Published:** 2015-07-25

**Authors:** Irja Marije de Jong, Frank Kupper, Marlous Arentshorst, Jacqueline Broerse

**Affiliations:** Athena Institute, VU University Amsterdam, De Boelelaan 1085, 1081 HV Amsterdam, The Netherlands

**Keywords:** Neuroimaging, Responsible research and innovation, Media-analysis, Technology assessment, Neurohype

## Abstract

Besides offering opportunities in both clinical and non-clinical domains, the application of novel neuroimaging technologies raises pressing dilemmas. ‘Responsible Research and Innovation’ (RRI) aims to stimulate research and innovation activities that take ethical and social considerations into account from the outset. We previously identified that Dutch neuroscientists interpret “responsible innovation” as educating the public on neuroimaging technologies via the popular press. Their aim is to mitigate (neuro)hype, an aim shared with the wider emerging RRI community. Here, we present results of a media-analysis undertaken to establish whether the body of articles in the Dutch popular press presents balanced conversations on neuroimaging research to the public. We found that reporting was mostly positive and framed in terms of (healthcare) progress. There was rarely a balance between technology opportunities and limitations, and even fewer articles addressed societal or ethical aspects of neuroimaging research. Furthermore, neuroimaging metaphors seem to favour oversimplification. Current reporting is therefore more likely to enable hype than to mitigate it. How can neuroscientists, given their self-ascribed social responsibility, address this conundrum? We make a case for a collective and shared responsibility among neuroscientists, journalists and other stakeholders, including funders, committed to responsible reporting on neuroimaging research.

## Introduction

Neuroimaging technologies have played a pivotal role in the boom of neuroscience research we have witnessed in the last three decades. In providing the opportunity to study the brain of a living subject without removing the skull, new ways were opened to study the physiology, anatomy and function of the human brain. This has resulted in new insights into how the brain is involved in pain, memory, movement, emotion, speech and thought.

Neuroimaging technologies have enabled the formulation of new research questions, beyond the traditional fields of the clinic and basic research, even yielding new interdisciplinary domains, such as neurolaw,^1^ neuromarketing^2^ and neuroeducation.^3^ However, the application of neuroimaging also warrants scrutiny of the relations it implies for our perceptions of self, human identity and the brain. This gives rise to questions about the biological underpinnings of who we are (Pickersgill et al. 2011; Farah and Wolpe 2004). Neuroscientists have been accused of the failure to distinguish between the brain and the mind, by reducing thoughts to specific patterns of neural activity (Tallis 2011). For emerging neuroimaging technologies, as for new technologies in general, ‘promise’ and ‘peril’ are intricately linked, as unanticipated effects in interaction with society ‘are not just possible but probable’ (Joss 1999; Hoffmann-Riem and Wynne 2002).

The question is, how to achieve this precarious balance between the promise and peril of neuroimaging applications? One answer is being sought in the emerging field of responsible research and innovation (RRI). RRI has gained considerable traction in science policies in the US, and EU since the turn of the millennium. It aims to guide science and technology-related research endeavours towards societal benefit, avoiding controversy and taking ethical and social considerations into account from the outset. It facilitates a process which is characterized by engagement with wider stakeholders. The interest in RRI seems to relate both to the successes and failures of science and technology initiatives in the past. Technology innovation can be a major driver of societal benefit. For example, new health research and technologies have contributed substantially to the health of individuals and communities worldwide in the previous century. However, research and technologies are also associated with environmental pollution, and to deep controversies, such as that regarding genetically modified organisms.

One could thus argue that neuroimaging is in need of RRI as an approach, and this seems to be recognized by two programs funded by the Netherlands Organisation for Scientific Research (NWO). The program “Brain and Cognition: societal innovation”^4^ funds a large part of the Dutch research in the field of functional neuroimaging relevant to the domain of justice and security. This program focuses on societal innovation and the inclusion of private or public societal partners. Secondly, our project “Neurosciences in Dialogue”, which specifically focuses on the societal embedding of neuroimaging technologies, is part of the funding program “Responsible Innovation”.^5^

Who is, or should be, taking the lead in this RRI approach? Interestingly, although RRI aims for a redirection of the natural sciences, the concept is pushed by the realm of policy, and natural scientists themselves seem not to be involved in negotiating its meaning. In previous research, we found that Dutch neuroscientists working with neuroimaging technologies were unfamiliar with the concept of RRI, and that they had difficulties in operationalizing this term for their practice (de Jong et al. 2015). We did find one concern that the RRI approach and the interviewed neuroscientists had in common: problems relating to neurohype and promises in science and research funding policy.

Emerging technologies are often accompanied by high expectations that are rarely lived up to, resulting in disappointment. As Brown (2003) has pointed out for the fields of biotechnology, e-commerce, stem cells and nanotechnology, expectations and disappointments have led to reputational damage and misallocation of resources. For scientists, disappointments pose a threat for public confidence in science and a loss of credibility for scientists. Scientists consulted in previous phases of our research expressed concerns related to the unrealistically high expectations for neuroimaging in the general public (in which they included policy-makers). And although neuroscientists expressed some concern about misrepresentations of the brain and neuroimaging in the media, they felt a social responsibility to prevent hyperbolic expectations or reduce hype by presenting an accurate and ‘balanced’ picture of neuroimaging technologies through the popular media (Edelenbosch et al. 2015; de Jong et al. 2015; Arentshorst et al. 2014).

In formal RRI conversations, it is also recognised that hype and unrealistic expectations pose challenges for the responsible governance of emerging technologies, and should therefore be mitigated in favour of more responsible constructions of technological futures (e.g. see Simakova and Coenen 2013). Therefore, despite the wide conceptual gap between neuroscience and RRI, common ground seems to exist in the shared aim of mitigating hype, in which neuroscientists then see the popular media as a preferred channel to achieve this.

**Fig. 1:**
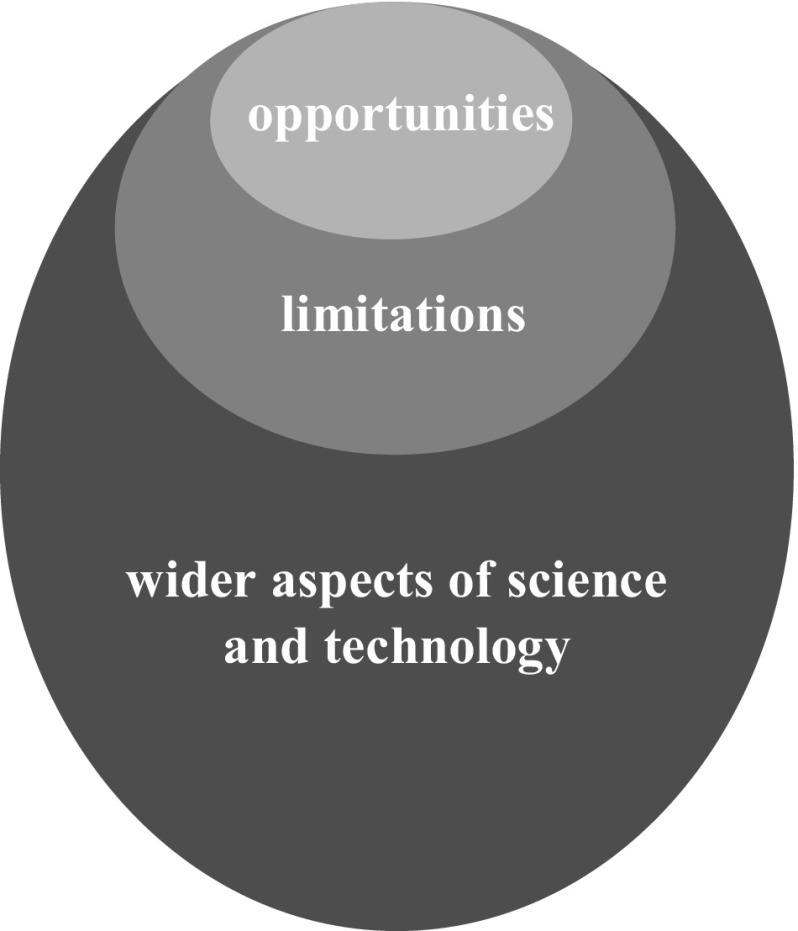
Opportunities, limitations and wider aspects of science and technology

Still, there remains a disjunction. Neuroscientists in our research argued for ‘balanced’ reporting, which to them means adequate attention to opportunities on the one hand, and limitations and caveats on the other (Seixas and Basto 2008). However, the emerging RRI community, emphasises consideration of the *wider* aspects of technology development, such as societal implementation, ethical acceptability and social desirability (see Fig. 1) (von Schomberg 2012). Addressing wider aspects of technology development encompasses, for example, reflection on the wider social context in which technology development is taking place. This relates to the recognition that technologies are not neutral and their development is not autonomous. Mutual shaping takes place between technology and society, in which social forces drive technological development in a complex fashion, while technology itself can shape history, culture and society (Williams and Edge 1996). Both social and technological context of technological development thus shape innovation choices, and as such constitute the form and use of technology in a contingent way.

If the mitigation of hype is an important element of RRI of neuroimaging, and the popular media is seen as an essential channel to realise this, can it be deemed successful? To what extent does the Dutch popular media actually present ‘balanced’ conversations on neuroimaging research to the Dutch public? Are limitations addressed besides opportunities, and are wider aspects of technology development also addressed? Whether balance is actually achieved also depends on the actors introduced in the storyline to voice opportunities, limitations and wider aspects, and whether all voices are attributed equal credibility (Eyck and Williment 2003). Furthermore, lexical choice (including the adoption of metaphors) also influences whether balance is achieved (Hansen 2011).

To answer this question of achieved balance, we performed an analysis of reporting on neuroimaging research in Dutch newspapers between 1992 and 2012. The media, and newspapers in particular, are a primary source of information regarding scientific and technological developments (Nelkin 1995; Rogers 1999; Becker et al. 2000; Best and Kellner 2001). This is especially so when technologies are in their early phases of development (Nisbet and Lewenstein 2002). Insights into media reporting offer opportunities for stakeholders, and neuroscientists in particular, to anticipate to the miscommunication of their findings to the public and hence to reduce the risk of unrealistic expectations and ethical or social concerns (Racine et al. 2010; O’Connell et al. 2011). We looked for the *opportunities* neuroimaging is thought to offer, as well as the *limitations*, and aspects relating to *ethical acceptability and social desirability* of the technology. Furthermore, as opinions of the opportunities and limitations of neuroimaging are informed by how the technology is understood, as well as how the object it measures is understood, we also decided to look for conceptual *metaphors* of neuroimaging and the brain in these articles. Lakoff (1993) describes a metaphor as a conceptual mapping from one domain to a different target domain, in which we draw from thought and experience to make sense of a complex reality. Metaphors are therefore not merely linguistic in nature but also cognitive and conceptual, as they aid in understanding complex phenomena in terms of a more structured concept. Finally, we looked at *who* introduced limitations and caveats in the texts: neuroscientists themselves, or other parties, such as the journalist, societal stakeholders or academics from other disciplines.

## Methodology

### Selection of News Articles

We generated a selection of Dutch newspaper articles using the LexisNexis Academic database (academic.lexisnexis.nl). This database contains full-text access to newspapers, both national and regional, dating back 30 years. We searched for Dutch-language articles published before 31 December 2012 containing at least one neuroimaging modality related keyword, such as ‘neuroimaging’, ‘brain scan’, ‘fMRI’, or ‘functional magnetic resonance imaging’, accompanied with occurrences of ‘science’, ‘research’ or ‘study’ within the same sentence and having ‘brain’ or ‘cognition’ in the body text. As this search query yielded too many sources (>3000 articles) for qualitative analysis, and to increase relevancy, we narrowed the search to occurrences of ‘brain’ or ‘cognition’ in titles and lead paragraphs and introduced the additional requirement of at least two occurrences of ‘brain’ or ‘cognition’, and of ‘science’, ‘research’ or ‘study’. During these subsequent steps, we checked for the randomness of the corpus, by exploring the proportion of articles per newspaper compared to the corpus of articles. To further reduce the sample size (>2000 articles), we subsequently selected the four national newspapers with the highest circulation rates (de Volkskrant, Trouw, NRC Handelsblad, de Telegraaf) for reasons of geographical coverage, as well as their apparent influence on other media coverage (Nisbet et al. 2003). To allow for ideological and geographical diversity, we also included a Christian national newspaper (Reformatorisch Dagblad) and five regional newspapers distributed over the Netherlands (De Gelderlander, Noordhollands Dagblad, Dagblad De Limburger, Dagblad van het Noorden, Brabants Dagblad). This resulted in a corpus of 398 articles. After removal of duplicates arising from the use of multiple keywords, republication of articles and exclusion of those articles that did not describe neuroimaging research outcomes, 306 unique articles remained.

### Coding and Analysis

All articles were read by two researchers (IMJ and MEA). A coding guide was created in consecutive steps. First, a random selection of 21 articles of the corpus was independently open labelled by IMJ and MEA. The two guides were discussed and a preliminary coding guide was constituted. The corpus of articles was independently coded with qualitative data analysis software (MAXQDA 11). The coded texts were discussed and amended when appropriate. A narrower focus was then discussed by IMJ, MEA and FK, and a final narrower coding guide was defined. All texts were then re-coded by IMJ. See Table 1 for categories of codes of the final coding guide.

**Table 1:** Coding guide

The final coding guide contained the following coding categories:
a) The newspaper and section in which the article was published
b) Date of publication, and word count
c) Reason for publication (news items or opinion item)
d) Neuroimaging research domain (healthcare, non-healthcare, boundary)
e) Insights (healthcare or non-healthcare, see Table 3)
f) Achieved or projected progress (healthcare, technological, scientific, other)
g) Limitations and wider aspects of science and technology
h) Nuance style (no nuance, disclaimer, correction, nuance, contestation, sensationalism)
i) Brain/scanning metaphor (see Table 2)

Conceptualization of phenomena (such as the brain or the scanner) can take place in several ways, with each being realized by different linguistic expressions (Lakoff 1993). For example the brain can be described as the “boss” or as a “slave driver”. Although linguistically different, both convey the image of the brain as a sentient being and of submission of the individual. Furthermore, metaphors can also be described in text. For example, when a brain is literally described as a “chatterbox”, this segment can receive the same code as a text segment in another document that describes a brain that continually talks. Although brain metaphors were coded as they emerged from qualitative analysis of the data, later these were clustered in coding categories as identified in previous work by Kupper and Drost (in writing), as these matched the findings here. See Table 2 for the coding categories of the conceptual metaphors of the scanner and the brain.

**Table 2:** Coding categories for metaphors, ordered by occurrence in individual articles

Scanning technology as	The brain as
*Illumination*	Object matter
Cartography	Sentient being
Evolution	Complex network
Revelatory	Embodied plasticity
	Functional device
	Self-organizing system

Although analysis was mainly qualitative in nature, we also calculated some statistical relations. During reading and analysing it was observed that the degree of nuance differed for neuroimaging research topics. Therefore, *p* values were calculated with two-tailed Fisher’s Exact Tests (Mehta and Hilton 1993) as a check for our observations.

## Results

### General Sample Characteristics

The selection contained 306 unique articles spanning the time frame of October 1992 to December 2012. No articles were found prior to October 1992 using the selection criteria. 77 % of the articles were published between 2005 and 2012. The number of words ranged between 58 and 2999, with a median of 524 words. One-third of the articles were categorized as brief stories (word count <350), and one-third were categorized as big stories (word count >1000). About half of the articles (n = 137) were found to be published in a general or unspecified newspaper section. The other half of the corpus, 131 articles, were placed in a science, knowledge or education section. The remaining articles were published under the heading of opinion (n = 7), healthcare and wellbeing (n = 12), religion, culture and philosophy (n = 10) and 9 articles are positioned on the front page. 24 articles (8 %) were written in response to earlier publication(s) (‘response articles’), others related to a specific event (such as a journal publication, conference, book release).

### Opportunities of Neuroimaging Research

Opportunities of neuroimaging research were related to different types of insights and progress (see Table 3). In 64 % (n = 195) of the texts, insights into a wide range of ordinary (non-pathological) thought, sensory experiences, behaviour and tangible social phenomena were described. Examples ranged from love, auditory processing, sleep, consumerism to aggression. This type of neuroimaging research mainly revolved around aspects of human identity and personhood.

**Table 3:** Coding categories for non-pathological brain insights, ordered by occurrence in individual articles

**Identified in more than 10 articles:**	Intelligence and learning
Brain development	Sleep
Lifestyle	**Identified in less than 10 articles:**
Language	Free will
Consciousness	Meditation
Justice/criminality	Religion
Mind reading	Gender
General brain insights	Sensory (auditory/visual/pain)
Neuromarketing/finance	Humans as social beings
Sex, lust and love	Gaming

A common theme can be discerned: Where we come from, who are we? What is our position in the whole? This may also be the answer to the question why there is much interest in brain research: You get new insights into who we are and why we behave as we behave. (Aleman and Dorst 2011, Reformatorisch Dagblad)

Among the different reported types of insights, healthcare insights were very prevalent in our corpus, as they could be recognized in 46 % (n = 143) of our texts.^6^ Examples are texts on mental disorders, neurodegenerative disorders, acquired brain injuries, behavioural disorders and clinical symptoms such as headache, pain and chronic fatigue.

The next two years the same four hundred people from Maastricht and Doetinchem (migraine patients and controls) will be retested. New MRI scans will also be made. A comparison should prove whether regular migraine attacks increases brain damage and whether that has functional consequences. (Scholtens 2007, de Volkskrant)

In 48 % (n = 147) progress was reported. As two neuroscientists described it in their own written article:

If these developments continue, many science fiction novels will soon have to be moved to the nonfiction section. (Poort and Roelfsema 2007, de Volkskrant)

Progress could be technological progress (n = 47, 15 %); or types of societal progress, the most prevalent being progress in justice and security (n = 15, 5 %), or in an economic/financial sense (n = 10, 3 %). However, the most encountered type of progress was clinical progress, with a prevalence of 36 % (n = 109). Importantly, clinical progress could be discerned in 60 % of the articles reporting clinical insights (n = 85 of a set of 143), but also in 14 % of the texts that reported only non-clinical insights (n = 22 of a set of 152).

Technological progress often related to the novelty and promises of new or existing neuroimaging modalities.

Finally, an MRI scan is made with ultra-modern equipment, yielding images that are infinitely better than the old scans. “On the old scans you see white matter, which contains the connections, lying as a fuzzy spot under the grey walnut-like cortex. The new MRI gives razor sharp colourful definition to that blurry spot. (van Oppen 2006, Brabants Dagblad)

Examples of achieved or projected healthcare progress were diagnostic applications or new options for treatment.

“We now examine how the machinery - the brain - works. Applications that can remedy the machinery, are then easily conceived of,” says the scientist. (de Volkskrant 2006)

A large part of our corpus addressed only opportunities. Of the 306 texts, 195 (64 %) only stated opportunities of neuroimaging research.

People think they choose a car brand or model on rational grounds, but a car is the most emotional product there is. With our scans we can now predict this emotion and the ultimate consumer choice with seventy percent certainty,” says [neuroscientist] Lamme […] “The standard today to put a car in the wind tunnel, will also apply to the MRI scan in the future,” he predicts. “So that the car will have the type of look that makes us happy. This will certainly make things look prettier. (Langenveld 2012, Dagblad de Limburger; Article with high word count)

This did not significantly differ for texts describing clinical or non-clinical insights and progress, however it was found significantly more within articles including the subtopic of healthy lifestyle (*p* = 0.0175) and significantly less for articles on locked-in syndrome (*p* = 0.0023). Furthermore, in articles with higher word counts, fewer articles were found that only presented opportunities.

### Limitations

Although in most of the texts of the corpus only opportunities were addressed, this was not the case for 36 % of them (n = 111). However, this does not mean that the texts within this part of the corpus all achieved ‘balance’. However, actors other than the neuroscientists themselves were often quoted when an attempt at balance was being made in these texts.

#### Disclaimers

In 48 articles (16 % of the entire corpus, and 43 % of the articles reporting limitations), limitations were mentioned in such a way that it functioned as a disclaimer. A typical example would be an article presenting insights and progress that also included a sentence on how more research and time was still needed to attain the said future. Articles on the topics of mind reading and consciousness were more likely to contain these kinds of disclaimers than other topics (healthcare and non-healthcare) (*p* = 0.0022 and *p* = 0.0048, respectively).

Although researchers rush to declare that it still takes some time before the discovery can be used to test testimonies of criminals for their veracity, the claim is nevertheless that the ‘brain lie detector’ is a lot more accurate than the current device unmasking lies by registration of physiological characteristics such as breathing rate, muscle tension and blood pressure. (Didde 2001, de Volkskrant)

#### Correcting Other Neuroscientists

In a small set, n = 5 (5 % of the articles reporting limitations), neuroscientists were given the floor to correct claims made by other neuroscientists. Usually this was done by emphasising wrong interpretations of the data by others, or methodological errors in the other’s research.

In Nijmegen, professor of biological psychology Ton Coenen subtly points out that the people from Twente first need to back up their claims, preferably with a living creature. “Only then can they make statements about it. For now, I would bet my money on our own research”. (de Volkskrant 2011)

Importantly, these articles did not contain quotes by neuroscientists in which they brought in limitations of their *own* neuroimaging research. Thus the impression remained that other individuals had messed up, and trust could be maintained in neuroimaging research in general.

#### Addressing Opportunities and Limitations

In 39 articles (13 % of the entire corpus, and 35 % of the articles containing limitations), we found a balance between opportunities and limitations. The regional newspapers were less likely to introduce limitations in their articles than their national counterparts (*p* = 0.0164, including the popular national newspaper “de Telegraaf”, and *p* = 0.0100 excluding “de Telegraaf”, for which the same pattern could be observed as for the regional newspapers). Free will discussions and articles presenting applications in the realm of justice and security were more often associated with articles reporting opportunities and limitations (*p* = 0.0023 and *p* < 0.0001, respectively). For example, there was emphasis on the limitations of neuroimaging research in distinguishing between cause and effect, or in producing individual outcomes.

“We do not know whether it is the chicken or the egg,” says neuropsychologist Prof. Dr. Peter Hagoort, director of the FC Donders Centre in Nijmegen, an institute for neuroimaging. Although the German researchers write that a defective nerve connection is a cause of stuttering, Hagoort remains cautious. “Maybe those abnormal nerves cause stuttering or maybe they are the result of the real cause.” (Dekkers 2002, de Volkskrant)

However, in seven of these articles in which the overall effect was a balance between opportunities and limitations, neuroscientists exclusively introduced the opportunities, whereas others (other academics, societal stakeholders or the journalist) introduced the limitations.

### Wider Aspects of Science and Technology

Wider aspects of science and technology were mainly presented in articles that either addressed both opportunities and limitations, or mainly contained contestation of neuroimaging research and applications. Interestingly, sensationalist topics and wording were more likely to be present in this set of articles (*p* < 0.0001).

Have you already had your brain stimulated transcranially? You really should. The stimulation of brains using magnetic fields is trending, indeed. The doctor shakes your brain cells locally awake by holding a powerful magnet to your head. (Keulemans 2008, de Volkskrant)

In 22 of 39 articles addressing both opportunities and limitations, wider aspects of science and technology development were also addressed. One such category was the difficult translation of insights to social and medical policy.

Policymakers often ask me [a neuroscientist], can we use these findings to improve schools? Or to address the juvenile justice system? At the moment, policy decisions are often made based on simplistic translations: [A certain brain region] does not yet function in young people, and therefore they do stupid things. But there is more to it, and those fine details should be considered in this type of policy decisions. (Korteweg 2012, NRC Handelsblad)

Medical diagnostics based on neuroimaging technologies were sometimes not deemed ready for implementation in medical policy because of the lack of a treatment option. Use of the diagnostic tool was then possible, but not ethically acceptable.

What to do with this knowledge? Should we offer every person over sixty an MRI brain scan each year, to bring silent infarcts to light? According to the researchers that only makes sense if there is a good treatment to prevent the onset of dementia after a silent cerebral infarction. However, such a targeted treatment is still lacking. (Kohler 2003, NRC Handelsblad)

Another theme was the application of neuroimaging in the context of justice. With insufficient insight into the accuracy of especially functional neuroimaging technologies, application within the domain of justice such as the courtroom was deemed undesirable, whereas this was not the case for application in the medical domain.

It is not free of consequences, you intervene in people’s lives: you’re basing far-reaching decisions on it, for example with respect to parole or even long-term imprisonment, while you don’t know how accurate the technology is.” What does fMRI ultimately say about behaviour? “You can have all kinds of dubious preferences and interests, but that does not mean that you act upon it. This is especially relevant for paedophilia, because research shows that there are people with a sexual preference for children who nevertheless still keep that under control. (ten Broeke 2011, Trouw)

But these examples were mere exceptions to the general observation that little attention is being paid to wider aspects of science and technology in which neuroscientists were quoted. As one science journalist noted in relation to social desirability:

What is completely omitted, is a debate about the desirability of this method of lie detection. Mohamed’s article promises more than it can deliver, but possibilities to put uncooperative suspects in the brain scanner are also explored elsewhere. The question is not whether it will ever be possible, but how to proceed íf it is possible - when lying is no longer possible because of the brain scanner. (van Maanen 2006, de Volkskrant)

Wider aspects of neuroimaging research were also found in articles in which the usefulness of neuroimaging research was altogether contested by other scientists (such as philosophers) and societal stakeholders (n = 18). Contestation was especially prevalent for the topic of measuring religion in the brain (*p* < 0.0001).

“It’s a bleak vision. We are not sitting here, talking to each other as just two brains,” says Bert Keizer, philosopher and nursing home physician. (…) Keizer also doesn’t exactly know what the soul is, but he is convinced that our spiritual life cannot be captured in brain scans. “Potatoes are material, the potato price is spiritual. They are inseparable, but you cannot say that potatoes consist of euros. (Giesen 2012, de Volkskrant)

Similar critiques were found in articles on free will/justice and security, and for articles on gender. In most of the articles contesting neuroimaging research, wider aspects were presented (n = 13). Neuroimaging research was claimed to be too reductionist, or too unilateral to provide insights into complex individual, social or societal phenomena. Neuroimaging studies were criticised for not acknowledging the wider context of the phenomena they were studying, or interpreting ambiguous data in a way to reinforce societal stereotypes. This was also seen to be combined with statements on poor methodological rigor in neuroimaging research, or doubtful interpretation of data.

### Metaphors

Metaphors generally surfaced only in one particular segment of text, to illustrate one specific phenomenon in a particular paragraph, rather than being used throughout the entire article.

#### Brain Metaphors

In order of prevalence, the brain metaphors we encountered were the brain as “object matter”, as a “sentient being”, as a “complex network”, as “embodied plasticity”, as a “functional device” and as a “self-organizing system”. Most frequently, the brain as encountered as *object matter*, in which material characteristics of the brain were stressed. For example, that it contains different types of “centres”, for pleasure, fear or pain, for example, or that it contains “wires” or “buttons”. A typical one related to “storage”.

The researchers also found that the more experienced the taxi driver, the greater is the area of the brain in question. London’s taxi drivers must first for about two years get to know the city before they get a license. The better they know the city, the bigger is the area in their heads where they ‘store’ the map. (de Volkskrant 2000)

The second most frequent metaphor regarded personalization of the brain, in the sense that qualities of an actor were ascribed to it. Two explicit examples for this metaphor type of the brain as a *sentient being*, was the brain being “chatty” and the brain as the “boss”. These were sometimes related:

[The neuroscientist], just like everyone else, has a “chatter box” in his head. With it, we collect knowledge on the outside world, communicate with others, and it commentates on everything we do. Very useful during the evolution, but it cannot be controlled by us. (NRC Handelsblad 2010)

Both these examples were found when non-clinical neuroimaging research, such as neuromarketing, was presented. However, the brain as a sentient being was also encountered in texts in which other neuroimaging research topics were encountered.

Less frequently found, the brain as *complex networks* argues that brain function arises from complex “networks” instead of specific singular spots or centres in the brain with specific fixed tasks.

We don’t think in terms of brain areas anymore, but in networks of areas, each brain function having its own network of sites. Areas function in different networks. Within such a network, an area can have a specialized function, but that is never an absolute specialization. Because it is also very active in very different networks. (Spiering 2005, NRC Handelsblad)

The complexity of the networks make the brain difficult to completely comprehend.

Also, texts that contained the metaphor of the brain as *embodied plasticity* generally conveyed that the brain keeps changing after it has reached maturity. Examples of this type of metaphors were the brain being “plastic” or “susceptible to training”.

Under the influence of those environmental, our plastic brains changes constantly. (Truijens 2011, de Volkskrant)

This was particularly found in texts on brain development, but also in articles on healthy lifestyle and related (cognitive) treatments.

The second least frequent metaphor, the brain as a *functional device*, emphasises the user-tool relationship. Examples are the brain as a “chemical plant”, as a “computer”, a “piano” or, as the next quotes illustrates, a “machine”.

Researchers are increasingly able to look into the engine room of our behaviour. (van Hintum 2012, de Volkskrant)

Least frequent was the metaphor focusing on the *self*-*organizing* quality of the brain, in which some connections are cut and others fortified in an autonomous fashion, yielding an optimized design of the brain. Examples are those of a “microcosm” or an “orchestra” without a clear structure.

Hagoort sees the brains as a huge orchestra, with brass players sometimes hitting the tympani, and a symphony joyfully alternated with a jazz piece, in which the double bass player of before is now suddenly playing bass guitar. “This orchestra with ever-changing line-ups and numerous versions has no conductor, and yet it plays no false note.” (Didde 2001, de Volkskrant)

#### Neuroimaging Metaphors

The most prevalent neuroimaging metaphor was that of “understanding by seeing”, in which two main components could be discerned, namely, the scan as “illumination” and the scan as “cartography”. The scan as illumination was comprised of expressions such as making parts of the brain “light up”, “making visible” and expressions of the subsequent recording of light in “photographs” or “pictures” of the brain.

MRI scan studies show that the brain lights up more strongly as the amount of money you can win in a lottery are higher. (de Volkskrant 2010)

Neuroimaging as cartography became apparent through wording such as “mapping” the brain, “tracking”, and producing a brain “atlas”.

Scientists can recognize brain patterns in the network activities of the brain, and subsequently map these. They can then use this ‘atlas’ to try to predict which brain networks are affected by the pharmacological mechanisms of new substances. (van Hintum 2011, de Volkskrant)

Occasionally, the neuroimaging metaphor of “understanding as seeing” was discounted. However, analysis of these instances did not yield a new (counteracting) metaphor.

“A brain image that expresses brain activity consists of 1 percent data and 99 percent statistics,” says neuroscientist Frank Leoné. A scan is not a “picture” and not comparable with a fingerprint or DNA, but a graphically displayed complex statistical calculation based on a number of assumptions and mutual agreements among experts. In addition, the coloured brain image in the newspaper is an average of a relatively small number of individual scans. (van Hintum 2012, de Volkskrant)

Other, less frequently encountered, metaphors emphasised either technological progress or particular properties of the scanning technology. With respect to the first, the metaphor of “evolution” was discerned, for example by talking about “refinement”, “advancement” and calling certain neuroimaging modalities “hyper modern”, compared to older scanning modalities.^7^ In a similar vein, in a small set of articles, the brain scanner was also portrayed as the “holy grail” or as the “object of envy”. With respect to the brain scanner as “revelatory”, it was found that neuroimaging technology was framed as revealing the future (“prediction”), or “uncovering” what we really think or feel, as illustrated by this article heading:

Brain scan uncovers fake orgasm. (Dagblad van het Noorden 2005)

## Discussion

The situation is fairly dismal for Dutch neuroscientists with their acknowledged social responsibility in terms of news media reporting of neuroimaging that prevents or at least mitigates hype. So far, they have not succeeded in their professed aim. And what’s more, they appear to be partly to blame.

Neuroimaging research was predominantly portrayed optimistically or neutrally in recent Dutch newspaper coverage. Neuroimaging research was presented as providing insights to understanding ourselves, or to gain clinical insights. Health progress, such as diagnostics and treatment had a prominent position. The image of progress was, for example, painted by favourably comparing (new) scanning modalities to more subjective methods or less advanced technological modalities, which was also found by Joyce (2008). Especially in Dutch regional newspapers and in one popular Dutch national newspaper (de Telegraaf), fairly little balance is present, as almost all articles only stress opportunities, sometimes in combination with a disclaimer. Articles presenting both opportunities and limitations or wider aspects of science and technology were considerably less prevalent. Addressing opportunities and limitations was found to be topic-related: healthy lifestyle was presented particularly optimistically, whereas reporting on justice and security was less optimistic than average. There were few critical articles as a counterbalance to the abundance of technologically optimistic articles. Also previous research on English language media coverage of neurosciences and neuroimaging showed, overall, optimistic and neutral points of view for neuroimaging use. This is mainly attributed to the failure of articles to address scientific, technical and ethical issues (O’Connell et al. 2011; Racine et al. 2006). The dominant frame position of healthcare in the neuroimaging research reporting is in line with previous research (Racine et al. 2006, 2010; O’Connor et al. 2012). It also concurs with the neuroscientists’ perception of a strong public interest in medical applications (Allgaier et al. 2013).

Ethical and social issues remained largely unaddressed. Moreover, neuroscientists themselves were often not the ones quoted when limitations or wider aspects of science and technology were presented: other parties were introduced for this, especially in the articles presenting contestation of neuroimaging research. It could be that potential alternative aspects raised by neuroscientists were neglected by journalists, as they may prefer other parties to tell that story, thereby favouring a more exciting story of polarized views. However, a recent study by Sumner et al. (2014) on health-related news showed that exaggeration in news is strongly associated with exaggeration in academic press releases. These researchers therefore recommend improving press releases produced by academics and their establishment, rather than pointing the finger at science journalists.

### RRI and Hype Mitigation

The outcomes of this study can serve as an eye-opener for neuroscientists wishing to mitigate neurohype through the media: contemporary reporting resulting from current interactions between the institutions of science and media is more likely to contribute to hype then to mitigate it. This finding corresponds with previous studies on other biotechnologies, which have shown narratives of hype to be typically present in much of the news media’s portrayals (Mulkay 1994; Conrad 2001; Caulfield 2004). Wider aspects of science and technology remain largely unaddressed. This is crucial, as an important theme within RRI is that of public deliberation on all aspects of research and technology development. However, this study shows that this role cannot be fulfilled by contemporary Dutch news media reporting on neuroimaging research, because of this absence.

For other emerging technologies it has also been observed that in times of controversy, more arguments are included of alternative social actors (such as non-government organisations), bringing other perspectives to the media (Flipse and Osseweijer 2013; Listerman 2010; Bonfadelli et al. 2002), than when an emerging technology is considered non-problematic (Romanach et al. 2015). This indicates that neuroimaging is generally not seen as a controversial technology at the moment, as social groups are not mobilized to get alternate points across. However, for neuroimaging this can be seen to vary per potential application. In articles on free will for example, and the related stance on responsibility for criminal acts, more critical voices were heard and wider issues addressed. But on this topic critical voices also belonged to the scientists themselves, not necessarily only to social groups. The gap between neuroscientists and the societal stakeholders was not always that wide. This could indicate that application in this domain is controversial among neuroscientists as well. However, in the particular case of free will, it could also be related to sensationalism. In a study by Allgaier et al. (2013), German neuroscientists put forward that reporting on neuroscientific experiments that challenge the existence of a free will was exaggerated and framed as a vast debate within neuroscience, when few are in fact engaged in this research topic. In the Netherlands, also little neuroimaging research is devoted to the matter of free will.

All in all, the current interactions between neuroscience and the media have yielded the body of articles described in this study and its associated trends. To prevent hype, the question is then: how can we transform the interactions to yield more responsible reporting on neuroimaging research, for all potential application domains?

### Sketching the Contours for Responsible Reporting in the Age of RRI

A transformation of the current interactions between the institutions of science and media, and between scientists and (science) journalists seems to be required. We therefore argue here for an experimental (small scale) construction of a neuroscience-media interface, as a locus to reinvent a *responsible* story of neuroimaging research and its prospective applications. Within such an effort, it is important not to treat hype rhetoric as a by-product, but to recognize it as a strategic resource for science, with respect to funding and recruiting allies, and for media in the constant battle for attention (Brown et al. 2000; Brown and Michael 2003; Caulfield et al. 2010). The good news is, that being part of the problem also means being part of the solution (Sumner et al. 2014).

#### What Story to Convey

Responsible reporting of neuroimaging research and ensuing applications incorporates wider aspects. Neuroimaging research does not merely consist of technological possibilities and barriers. Wider aspects entail historical, cultural, social, societal and normative connotations of neuroimaging research and of application options. Decisions are constantly being made in the research and technology development process, and these have political aspects and are connected to economic interests. What is the rationale for the types of neuroimaging research that are being performed, and not being performed?

#### Rethinking News Value

Attention should be paid to the ongoing process of neuroimaging research as a collective effort and the different stances taken in it, instead of to outcomes of demarcated single studies. In our study, we observed an abundance of news articles in which the novelty was a new publication in a renowned journal of a single neuroscience study. The (modest) new insights are then positioned as a critical step towards promising futures. The repetition of this link to promising futures can fuel hype and is under-critical of the multitude of factors influencing the translation of scientific insights to an implementation context. Within the news media structure, how can “news value” for science journalism be operationalized in such a way that it allows for responsible reporting? With respect to the wider aspects of science and technology, the exploration of long-term trends are (at least) of equal importance as the introduction of novel issues. Other barriers to responsible reporting should also be discussed, for example time pressure and the struggle for attention in the media on the one hand, and the role of hype in access to research funding and the credibility of scientists on the other hand (Allgaier et al. 2013; Williams and Clifford 2009). How do regional newspapers fit in, with respect to telling the story of neuroimaging research? Do they have specific barriers, for example with respect to access to specialized science journalists? Citizens do not necessarily read national newspapers.

#### The Neuroscience-Media Interface

To address this multitude of factors relating to neuroimaging research and applications, it would be wise to organize interactive, transdisciplinary meetings at the boundary between neuroscience and media that can enable a wider conversation on the story of neuroimaging research and application options from multiple perspectives. With scientific, technological and societal factors being in continuous flux, these meetings should be recurrent, instead of one-off. Moreover, this will enable the growth of a new practice at the boundary of neuroscience and media. Key players from the institutions of neuroscience are not only the individual scientists themselves, but also public relations professionals employed by research organizations, research funders and science policy-makers. Similarly, not only science journalists should be involved in these discussions, but also news editors and press release officers. The inclusion of the latter group is important considering the decline in the number of specialist science reporters and the simultaneous growth of a community of press officers (Williams and Gajevic 2012). For the consideration of the wider aspects, professional stakeholders (such as prospective end-users) can be included, but also other types of scholars, especially social scientists or humanities scholars. A study by Peters et al. (2008) underlines the need for the inclusion of non-science and non-media actors, as there is the potential for “too much control by the scientific community of media coverage about it, as well as that for too much media influence on inner-scientific processes”, thereby positioning science-media interactions in a too positive light. The promotion of multi-directional communication between actors from science, social science, media, the public as well as other stakeholders has also consistently been advocated by others to avoid a misunderstanding of neuroscience, as well as an enhancement of scientific literacy (Illes et al. 2010; Racine et al. 2005; Samuel and Kitzinger 2013).

Some experimentation with new collaborations between science institutions and media are already taking place. The founding of Science Media Centres in the UK is a notable example (Fox 2006; Haran 2012). However, there is a danger of these initiatives turning into an “instrument to secure science’s license to practice” (Rödder 2014). This underlines the importance of also including scholars from the social sciences or humanities, and the possibility to have them facilitate such hybrid meeting places. Similarly, Samuel and Kitzinger (2013) have argued for the inclusion of social scientists’ perspectives in scientific press releases on neuroscience research which could avoid churnalism (mere reproduction of scientist perspectives offered in the press release).

Such an interface could provide the opportunity for neuroscientists to get acquainted with and be encouraged to take up the wider social and ethical considerations, not only in their communication, but also in their work. In the best case scenario, in accordance with RRI as an ideal, societal values would serve as an inspiration, rather than as mere restrictions. As Flipse et al. (2013) have also previously stated that through the integration of social and ethical aspects in collaborations, more and better research options, goals and priorities can be generated, which could otherwise have been overlooked.

#### A Role for Metaphors

Metaphors can be helpful in this sense-making effort, as they have an explanatory and communicative capacity (Kueffer and Larson 2014). The use of metaphors is however fraught with ethical considerations in itself. For example, within an open dialogue the goal should not be to convince an audience of a certain stance but to allow for inclusive communication and mutual understanding. Moreover, metaphors can have socio-political consequences, as they can influence what type of research is seen to be relevant to engage in (Kueffer and Larson 2014). The work by Nerlich et al. (2002) on metaphor use in the Human Genome Mapping Project, for example, showed a strong link between the story frames in news reporting and the metaphors used by scientists and politicians to steer public discourse towards public euphoria.

Multiple types of metaphors relating to the brain were uncovered. Neurohype can be sustained by metaphors that favour oversimplifications. Simplification of science due to the use of metaphors has been alleged to take place by Knudsen (2005) and Christidou et al. (2004). In our study, oversimplification occurs when complex phenomena such as religious experiences are reduced to activities in certain ‘spots’ in the brain, which is found within the category of the brain as *object matter*. The brain as a *functional device* can also be prone to this oversimplification, for example the brain as a machine or a computer. Some of the more frequent instances of the brain as a *sentient being*, the brain as the boss, can induce the view that behaviours and feelings are beyond one’s control. The brain can then be seen to determine personal characteristics and to be the locus of difference between people. This is less likely for the brain as *complex networks*, as *embodied plasticity*, or as a *self*-*organizing system*. The brain as complex networks, and the brain *being* plastic, opposes a reductionist image of the brain, as it undermines the idea of the brain operating though fixed spots with fixed functions, and capitalizes upon the idea that one’s brain, behaviour and health can be modulated. However, the linguistic terms used here, plasticity and networks, matches the jargon of the scientists themselves more closely, and hardly seem to be chosen for their explanatory or communication function for the intended audience; the general public. It is therefore questionable whether these metaphors are equally powerful.

One particularly strong metaphor was found for neuroimaging technologies, namely that of “understanding by seeing”, with the two sub-categories of neuroimaging as *illumination* and *cartography*. Its abundance is probably related to the wider socio-technological shift towards visualisation (Joyce 2008). One often seen example of this metaphor was the description that certain parts of the brain “lighted up” in the scanner. This metaphor was also found in media studies on neuroscience reporting by Samuel and Kitzinger (2013), O’Connor (2013) and Joyce (2008). Both sub-categories have connotations of precision and detail, and of fairly straightforward representations. These metaphors shield aspects of manipulation in the process of producing (as opposed to acquiring) the image of “the” brain. We found instances where oversimplification—as a result of the adoption of certain metaphors of neuroimaging—was contested in an effort to achieve balanced information on neuroimaging research. However, we were unable to find metaphors that conveyed these other ignored aspects of neuroimaging and responsible reporting might thus require an effort to do so. There is also the issue of assessing whether brain metaphors in the categories of complex networks, embodied plasticity and self-organizing systems are powerful enough to counteract those that suggest a reductionism of the brain. The aim of RRI of preventing and mitigating hype by addressing the wider aspects of neuroimaging technologies could thus benefit from the deployment of multiple metaphors in news media, either within a single newspaper article, or across a set of individual newspaper articles (Raymond et al. 2013). The proposed neuroscience-media interface could be a site for consideration which wider aspects of neuroimaging technologies are covered by what metaphor, as well as provide a locus for the conception of new metaphors for those considerations that are systematically ignored. For example, can a suitable metaphor be thought of to convey how neuroimaging images are achieved through manipulation of data, to counteract the idea of direct representation? Furthermore, as this study indicates, reporting can vary significantly per application topic. Therefore, it can depend on the neuroimaging research topic if certain metaphors are more salient than others with respect to hype mitigation. Whether that is the case, is also something that can be discussed within such a neuroscience-media interface. When (new) metaphors have been made explicit in such a site, audience research or participatory processes including the general public could be used for the assessment and improvement of metaphors.

### What It Could Look Like

So far, we have suggested a possible agenda for the proposed neuroscience-media interface, to develop a more responsible narrative for neuroimaging research and how metaphors can play a role in telling the story. But then what? What do we see as the potential concrete *output* of such an initiative, which could possibly turn the situation around?

We envision this initiative could develop outlines for multiple rich and deep storylines on neuroimaging research. These can be drafted as invitations for (science) journalists (and/or others) to submit proposals for each particular storyline. These would then be reviewed and selected by an editorial team. This editorial team would aim to represent and allow the expression of a range of views, using a variety of media and stylistic approaches, rather than to put forward an official line that is free of dissent. A fee would be paid for working out the complete version. These stories should then not only be published in national newspapers, but also in regional newspapers, where the least ‘balanced’ stories were found. Additionally, the initiative can draft similar invitations for further neuroimaging and brain metaphor research, for assessing or improving metaphors. These metaphors can then also be included in future storylines. Moreover, the initiative can decide to support neuroimaging researchers who (want to) write science blogs, as many readers interested in science not only turn to print media, but also look for it online (Blakeslee et al. 2012).

This initiative and its activities will require funding. As (governmental) research sponsors and neuroscience research institutions themselves both benefit from the prevention of hype and ensuing disappointment, part of the research budget could be used to fund the activities of this initiative.

With the ethics of science communication attracting increasing attention (Murcott 2006), such an effort is sorely needed for neuroscience as well as for other sciences. In principle, the framework outline here could also be used for the improvement of science reporting on other scientific and technological domains: both for those already existing as for those newly emerging. However, considering the growing recognition of the importance of upstream deliberation (Wilsdon and Willis 2004), emerging scientific fields and technologies are in particular need of collaborative spaces, as described here. The creation of a collaborative space consisting of neuroscientists and science journalists in conjunction with news editors, social actors and social scientists could function as a worthwhile test-case for other (future) emerging fields of science and technology.

### Methodological Considerations

We only included articles that report about neuroimaging research, thereby excluding those articles reporting about neuroimaging without references to research. Generalisation of our results beyond the domain of neuroimaging research without additional analysis of media articles without references (i.e. opinions, columns, and etcetera) is therefore not possible. Furthermore, our corpus of articles contained a minority of articles that show more negative points of view regarding neuroimaging research and its outcomes. Given that other similar studies also concluded that the number of articles that present a more negative or pessimistic point of view were scarce (Racine et al. 2006; O’Connell et al. 2011), our results are most likely representative of the current state of coverage of neuroimaging research in Dutch newspapers.

## Footnotes

1 Neurolaw investigates problems at the intersection of neuroscience and criminal justice. See for example: http://lawneuro.org/blog/category/neurolaw/.

2 Neuromarketing studies how human brains respond to advertising and brands. See for example the website of the Dutch firm Neurensics: www.neurensics.com/en/.

3 Neuroeducation links education and neuroscience in an effort to improve learning and instruction. See also Ansari et al. (2012).

4
Www.hersenenencognitie.nl/contents/1038?locale=en.

5 This project focuses on the responsible development and use of neuroimaging in three application domains, being health care, education and justice & security, and involves researchers and societal stakeholders in an interactive learning process to stimulate developments towards shared desirable applications with few, or at least manageable, negative impacts.

6 Because of overlap, the sum exceeds 100.

7 An example is the previous quote in Sect. 3.2 from the article by van Oppen (2006).
